# Point: Incident Exposures, Prevalent Exposures, and Causal Inference: Does Limiting Studies to Persons Who Are Followed From First Exposure Onward Damage Epidemiology?

**DOI:** 10.1093/aje/kwv225

**Published:** 2015-10-26

**Authors:** Jan Vandenbroucke, Neil Pearce

**Keywords:** dynamic populations, epidemiologic methods, incidence rate, incident exposure, left truncation, prevalent exposure, right censoring, study design

## Abstract

The idea that epidemiologic studies should start from first exposure onward has been advocated in the past few years. The study of incident exposures is contrasted with studies of prevalent exposures in which follow-up may commence after first exposure. The former approach is seen as a hallmark of a good study and necessary for causal inference. We argue that studying incident exposures may be necessary in some situations, but it is not always necessary and is not the preferred option in many instances. Conducting a study involves decisions as to which person-time experience should be included. Although studies of prevalent exposures involve left truncation (missingness on the left), studies of incident exposures may involve right censoring (missingness on the right) and therefore may not be able to assess the long-term effects of exposure. These considerations have consequences for studies of dynamic (open) populations that involve a mixture of prevalent and incident exposures. We argue that studies with prevalent exposures will remain a necessity for epidemiology. The purpose of this paper is to restore the balance between the emphasis on first exposure cohorts and the richness of epidemiologic information obtained when studying prevalent exposures.

***Editor's note:****Counterpoints to this article appear on pages 834* *and* *840*, *and a response appears on page 846.*

The notion that epidemiologic studies should start with participants that are observed from initiation of exposure onward (1) and that this is necessary for causal inference has recently received wide support (2–6). In contrast, studies that enroll people with “prevalent exposures” are considered to be prone to selection bias (7). The “first exposure” approach received support because of observational research wrongly indicating that combined hormone replacement therapy (cHRT) protects against myocardial infarction. It is also supported on theoretical grounds rooted in counterfactual thinking (see below). Conversely, for over a century epidemiologic studies have involved prevalent exposures, and their results have largely stood the test of time. In this paper, we argue that studies (cohort or case-control) that estimate incidence rates and rate ratios (hazard ratios) in dynamic populations (8, 9) involving a mix of prevalent and incident exposures are necessary, and that universal application of the first exposure principle would be detrimental to epidemiology.

## PRELIMINARY CONSIDERATIONS

We will first consider some background issues about types of evidence, populations, exposures, follow-up, and patterns of hazard ratios over exposure time.

### Types of evidence used in causal inference

The first exposure principle is supported by theories of causal inference that hold that causality can only be assessed by either a randomized trial or an observational study that closely mimics a randomized trial. In a randomized trial, follow-up usually starts from the initiation of an intervention (it could be conducted in a population with previous/prevalent exposure/treatment, but the focus would be on the effects of the new (randomized) exposure/treatment). By analogy, the effect of an exposure in an observational study can only be fully and validly assessed if follow-up starts from first exposure. The first exposure principle is implicit in the theory of potential outcomes and counterfactual thinking (10, 11). Recently, the principle has been made more explicit (2, 12, 13).

This view of causal inference is different from that in Austin Bradford Hill's causal considerations that may “…  help us to make up our minds on the fundamental question—is there any other way of explaining the set of facts before us, is there any other answer equally, or more, likely than cause and effect?” (14, p. 299). The evidence used in such considerations includes analytical studies that may include prevalent exposures (having hypertension, being diabetic), as well as evidence from time trends, ecological analyses, and biological and mechanistic insights. Although some types of evidence may be better than others for a specific hypothesis, there is usually no definitive single study or study design that answers the question of causality on its own. Moreover, randomized controlled trials (or observational studies which mimic such trials) may be regarded as the (hypothetical) “gold standard” for many research questions, but there are other important research questions for which it is difficult to conceive of a definitive randomized trial even in theory (e.g., the health effects of climate change, or macro-level economic policies).

### Types of populations

Populations can be closed or open, and the distinction depends in part on the time axis used (15). For example, a population of people who have used a particular medication would be “closed” if time is defined as time since starting medication but would be “open” if time is defined as calendar time. In addition, populations may be regarded as dynamic if people can join, leave, and rejoin (e.g., the population of London during the year 2014) or may be regarded as closed, or fixed, if this is not permitted (e.g., follow-up of everyone living in London on January 1, 2014).

In the current context, the key distinction is whether the population is open to entry after the exposure under study begins and open to exit before the outcome under study occurs (S. Greenland, University of California, Los Angeles, personal communication, 2013). The first part of this criterion involves the distinction between a study of “incident exposures” in which follow-up starts from (or before) the time of first exposure and a study of “prevalent exposures” in which follow-up may start after first exposure. This leads to left truncation (missingness on the left). Almost all studies also involve right censoring (missingness on the right), because there will be a proportion of participants whose length of follow-up is restricted.

**Figure 1.**
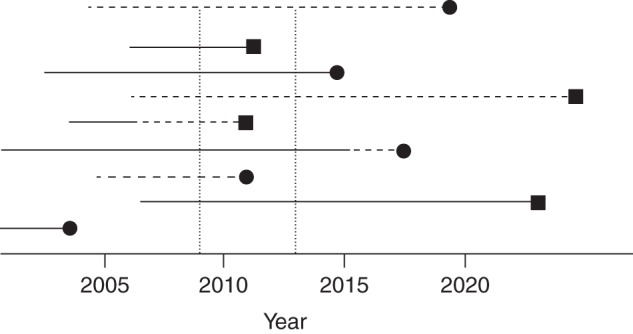
A hypothetical population that is dynamic with respect to calendar time. The period of observation falls within the 2 vertical dotted lines: the 4-year period 2009–2012. Solid horizontal lines (—) represent exposed time experienced by a person in a dynamic population; dashed horizontal lines (----) represent unexposed time; black squares (▪) represent incident cases; black circles (•) represent loss to follow-up.

Figure 1 shows a population that is dynamic with respect to calendar time: Participants enter at different calendar time points and are either exposed or unexposed, or they switch exposures over time. The population is studied for the 4-year period 2009–2012, and the investigators will have information only on disease incidence during this period (although they may be able to collect information on exposures from previous periods).

**Figure 2.**
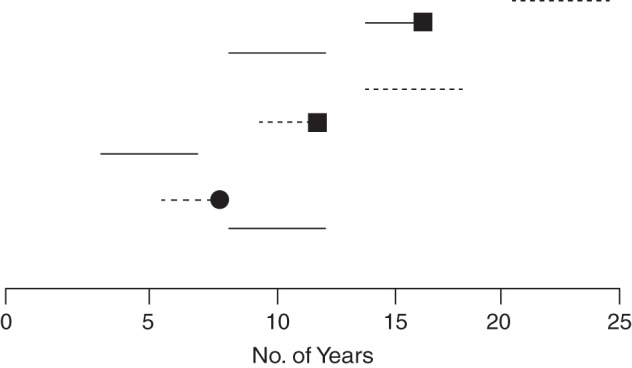
Illustration of how the observation period of the dynamic (with respect to calendar time) hypothetical population in Figure 1 includes just some of the information from the corresponding fixed (with respect to start of exposure) cohort study in which follow-up would have started with first exposure or the corresponding date in the nonexposed. The time axis is exposure time and starts at initiation of exposure or beginning of follow-up in nonexposed persons (time 0). Solid horizontal lines (—) represent exposed time experienced by a person in a dynamic population; dashed horizontal lines (----) represent unexposed time; black squares (▪) represent incident cases; black circles (•) represent loss to follow-up.

Figure 2 shows how this selected period of follow-up from the 4-year calendar time period then matches up with the information that would have been obtained in the corresponding fixed (with respect to start of exposure) cohort study in which all participants would have been followed from first exposure or the corresponding date for the nonexposed. The key feature is that each study participant is at most followed for 4 years. For some, this is the 4 years following the start of follow-up (exposed or nonexposed), but for most it represents a different period (e.g., years 7–11 after first exposure or start of follow-up). There is “left truncation” (delayed study entry at different times during follow-up); if the entry at different follow-up times is not taken into account, the data would represent a mix of the information that would have been obtained from different follow-up periods in the full fixed cohort study, and selection bias may occur (1, 2). However, this phenomenon is well known in the clinical epidemiologic literature (16–18), as well as in the occupational epidemiology literature (19), and standard analytical solutions exist (e.g., by calculating life tables with left-truncation, or by calculating person-years of exposure in different categories of follow-up time) (19–21).

### Types of exposure

Irrespective of when people are recruited into a cohort (or identified as cases and controls in a case-control study), information can be collected on previous exposures. Thus, prevalent exposures can be classified by using information on current exposure, past exposure, or cumulative exposure, and incident exposures can also be classified the same way once follow-up has proceeded. For example, for cigarette smoking and lung cancer, cumulative exposure may be most important; this can be assessed at each person-year of follow-up irrespective of when smoking started. On the other hand, for hormone replacement therapy and myocardial infarction, the recently initiated exposures may be most relevant. Finally, there are exposures that are “on-off” phenomena or exposures that are completely transient, which also determines the type of follow-up that is needed.

### Types of follow-up that are needed

The relevant follow-up period will differ according to the context. For example, in a study of venous thrombosis, follow-up of a person who was using oral contraceptives at age 25, had used the pill for 6 years, continued to use the pill for another 13 years, and then stopped gives useful information for several time windows of use/follow-up. Follow-up might start with prevalent use and end at most 3 months after stopping. This approach would be adopted mainly because the risk of venous thrombosis with oral contraceptives is expected to be an “on-off” coagulation effect, with perhaps an early peak, but followed by a sustained increased hazard ratio until shortly after cessation of use (22–24). On the other hand, in a study of oral contraceptives and breast cancer, follow-up should continue for many years after stopping use. In the first instance, the effect is due to the “drug in the blood” and stops when the drug is no longer present; in the second instance, the effect is presumably due to lasting cellular alterations.

Exposure-disease associations in case-crossover studies are often an extreme form of expected on-off effects: They are transient, more or less instantaneous, and completely reversible (e.g., coffee drinking and myocardial infarction (25), cell phone use and car accidents (26)).

### Patterns of hazard ratio by time since first exposure

**Figure 3.**
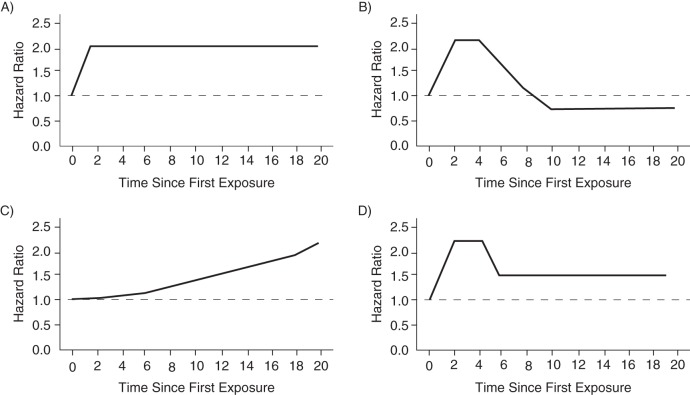
Some possible patterns of hazard ratios by time since first exposure in a hypothetical population. Time axis is arbitrary and can be minutes, days, months, or years. A) Hazard ratio is constant by time since first exposure (latency period can be short); B) initially increased hazard ratio followed by reduced hazard ratio; C) increasing hazard ratio by time since first exposure; D) initial increase, followed by lower but still elevated hazard ratio.

Figure 3 shows 4 general patterns of hazard ratios by time since first exposure. They can be viewed as daily hazard ratios from date of first exposure onward (i.e., the ratio of daily hazard rates or incidence rates between exposed and nonexposed, analogous to the theory of the Cox model). In Figure 3A, the hazard ratio is constant from first exposure onward, after an initial latency period. If the time axis was days (or even minutes), this pattern would apply to transient and on-off exposures whose effect is present as long as the exposure is present. Figure 3B shows an initially increased risk followed by a subsequently reduced risk, a pattern that was found for cHRT exposure and myocardial infarction over years of follow-up (2, 27–29). In Figure 3C, the hazard ratio increases with duration of exposure; the findings for cHRT and breast cancer are compatible with this pattern over years of follow-up (27, 30–32). Figure 3D shows an initial peak followed by a sustained increased risk; this pattern was seen for cHRT and venous thrombosis (33, 34). The consequences of left truncation are different for these different exposures.

## THE PROBLEM OF LEFT TRUNCATION (MISSINGNESS ON THE LEFT)

### Hormone replacement therapy and myocardial infarction

The potential problem with studying prevalent exposures has been highlighted in the cHRT controversy. Observational studies showed a consistent overall decrease of myocardial infarction among users of cHRT. In contrast, in randomized trials cHRT increased the risk of myocardial infarction in the first years of use, but the hazard ratio was subsequently decreased (sketched in Figure 3B) (35). This apparent contradiction was solved by the realization that the observational studies started with current users (35), who were contrasted with never users or with past users. At the start of follow-up, the current users were mostly past the initial period with elevated risk; they showed a decreased hazard ratio of myocardial infarction that corresponded to the findings of the randomized trials *after* the first few years of use (28, 29, 35, 36).

Two different analytical solutions exist. First, analogous to the reasoning about left-truncation when discussing types of populations, analyses in the observational part of the Women's Health Initiative study included study participants with prevalent exposures, stratified on duration of cHRT use (synonymous with time since first exposure) at enrollment, and showed that for coronary heart disease the observational study findings were consistent with those of the Women's Health Initiative randomized trials (36). Second, an analysis of the observational Nurses' Health Study compared incident users, based on counterfactual thinking (see above): It excluded prevalent users, identified new (incident) users of cHRT from each biannual examination, and analyzed these data as a sequence of imaginary successive randomized trials; this also made the findings for coronary heart disease consistent with those from the randomized trials (29). The first solution was particularly suited for analyzing the Women's Health Initiative observational data, as there were few women who initiated cHRT during the follow-up; the second approach was appropriate for the Nurses’ Health Study as there were ample women initiating cHRT during successive biannual periods (36). The 2 methods were proposed in different contexts, but their underlying approaches are consistent and complementary. There is debate whether a similar biasing effect of studying prevalent users may exist for observational studies of the effects of statins (refer to Web Appendix 1, available at http://aje.oxfordjournals.org/).

### Confounders that change after initiation of exposure

Confounders and other covariates (e.g., mediators) may change over time, and this may be caused by previous exposure. This phenomenon can exist in all follow-up studies of a fixed cohort. An “old” cohort (i.e., the latter part of follow-up) may differ from the baseline with respect to these covariates. Analyses that adjust for variables that are affected by exposure may produce bias (7). This may be important in some circumstances (e.g., in clinical and pharmacoepidemiologic studies of beneficial effects) when therapy changes in the course of follow-up and where confounding by indication plays a large role (i.e., the indication for treatment may have changed). It may be less important in studies of adverse effects, in which the risk factors for adverse events are different from the reasons for exposure (37) (refer to the example about nonsteroid antiinflammatory drugs below), or when the exposure is a recurrent transient phenomenon such as cell phone use while driving, or coffee drinking (25, 26).

### The exclusive solution: study follow-up from beginning of exposure

It has been argued, on the basis of the cHRT experience, that all “good” exposure-disease studies should start follow-up from the start of exposure (3, 4, 38–40). Moreover, starting from inception of exposure enables us to capture the life-course effects of exposure. Studies of prevalent exposures are weighted more towards participants who started exposure at some time in the past. This is not a major problem—in the sense that it will not put epidemiology completely on the wrong foot—if the hazard rate is reasonably constant across time periods since the first exposure (Figure 3A), is slowly increasing (Figure 3C), or if an early peak is followed by a sustained risk (Figure 3D). However, in the cHRT and myocardial infarction example (Figure 3B), the cohort studies effectively placed greater weight on the later time periods where the rate ratio was less than 1.0 and less weight on the earlier time periods where it was greater than 1.0. Although limiting the analysis to persons followed from first exposure onward solved this problem in 1 study (29), an analysis by duration of exposure (time since first exposure) (28) addressed the problem equally well and showed essentially the same findings (36).

### An inclusive solution: the period analysis life table

Methods to account for left truncation are not new (20). In oncology, a period analysis is used because the prognostic information for the near future is better with period analysis than with full cohort analyses (41). Suppose that an investigator wants to know the 20-year survival of persons who have undergone colorectal surgery for colon cancer. In a regional (or country wide) cancer registry, all persons are identified who are alive after such surgery on January 1, 2009, and thereafter all new patients who undergo the same surgery are enrolled; all are followed until December 31, 2012. Follow-up is at most 4 years. However, these 4-year periods represent different time windows since the start of exposure for different patients. For each of these time windows, incidence rates of death are calculated, which are then transformed to cumulative incidences, and a life table can be calculated (analogous to our Figures 1 and 2).

The 20-year cohort experience that is reconstructed is *not* the same as the cohort experience that is obtained when starting follow-up with everyone who had surgery 20 years ago. The latter life table is of little interest for future patients, since it concerns surgical and medical practices that may not apply in the future. Conversely, the follow-up experience of those in their 16th–20th year of follow-up in the 2009–2012 time window may be of less interest to persons who receive their surgery in 2009–2012 because their survival is most likely going to be different 20 years in the future, but it still has the advantage of representing the recent state of health care. The most recent (in calendar time) follow-up experience may be the most valuable for decisions about future health care (41, 42). The problem of left truncation in a life table was already described and solved several decades ago in the statistical literature (refer to the reports by Turnbull (42, 43), Detels et al. (44), and Hsia et al. (45) for an overview of the older literature), but solutions were sometimes reinvented by shrewd clinical investigators who faced the problem in studies on the prognosis of transient ischemic attacks (18), of colon cancer risk in patients with colitis (16), and of multiple sclerosis (17).

Similar analyses are routinely used in cohort studies and case-control studies based on dynamic populations, wherein hazard ratios or odds ratios from case-control studies are often presented for different strata of duration of exposure. Hernán (2) has proposed that it might be more insightful to present such findings as cumulative risks, rather than as an array of odds ratios or hazard ratios, as one can string together the incidence rate ratios for successive duration of exposure/follow-up windows (as in the period analysis life table). Refer to Web Appendix 2, including Web Figure 1, for an example.

### How generalizable is the problem?

How important is the cHRT and myocardial infarction phenomenon for epidemiology? Severely biased findings for the association of cHRT with myocardial infarction were derived in epidemiologic studies, because the hazard ratio completely reversed over the follow-up time (sketched in Figure 3B). However, the same studies with prevalent cHRT users did *not* produce any grossly wrong estimates for other outcomes: The findings for venous thrombosis or pulmonary embolism, breast and colon cancer, or strokes were all in line with the findings of randomized trials (27, 36). For venous thrombosis, there was an initial peak (as sketched in Figure 3D), followed by a sustained risk that was quite similar in both randomized trials and observational studies (33, 34). For breast cancer, both observationally and in the randomized trials, there was a slow increase in cumulative risk (31), as well as a suggestion for an increasing hazard ratio (as sketched in Figure 3C) over exposure time (27, 30–32). Thus, all these results were in the same direction for randomized trials and observational studies and were numerically similar in their order of magnitude. Moreover, separate analyses for duration of exposure showed the pattern of the hazard ratios over time. None of these other outcomes led to a clear selection bias problem because of the use of prevalent exposures.

The reasons why the hazard ratios of myocardial infarction went below unity in long-term users are not clear. Depletion of susceptibles has been proposed (2), but this may not be a complete explanation (Web Appendix 3).

A follow-up study of different nonsteroid antiinflammatory drugs and the occurrence of myocardial infarction as an adverse effect (46) is an example of the combination of the best of both worlds, as it was possible to do sensitivity analyses by comparing new users with prevalent users and to assess the effects of the potential selection bias from the latter. There was an early peak following first exposure, but the main analyses yielded similar findings just using the overall incidence rate ratios. Thrombotic phenomena with nonsteroid antiinflammatory drugs are an example of a “drug-in-the-blood on-off” phenomenon, where associations are present as long as the drug is used, which allows that parts of the follow-up are started during current exposure and are stopped at the cessation of exposure. In addition, the data were obtained in a period wherein the problem of myocardial infarction occurrence with different types of nonsteroid antiinflammatory drugs was not yet known, so any reason for prescribing or changing prescriptions would have been the result of noncardiovascular factors (e.g., stomach complaints), and bias from confounders changing over time would be unlikely (37).

The iconic follow-up study of smoking and lung cancer by Doll and Hill (47) started with prevalent exposures for smoking. The “current versus never” contrast yielded very large risk ratios, and duration of smoking was a strong risk factor in all analyses. There was no risk reversal during follow-up. On the contrary, risk continued to increase strongly over exposure time. Even after cessation of exposure, continued increased risks existed that may gradually return to background levels (48). This phenomenon is not unusual. In many studies of chronic environmental or occupational exposures and cancer (e.g., nickel and nasal cancer, asbestos and mesothelioma), it is standard to include both prevalent and incident exposures (19). Such studies start with the current employees of the factory/industry on a particular day in the past and then add in new workers who joined subsequently, and it is possible to have separate incidence rate calculations for different time periods since first exposure. This allows us to have all the information that one could obtain from a study of incident exposures—and much more quickly, and in larger numbers—analogous to the period life table analysis. Omitting prevalent users would lead to a considerable reduction in study size and would increase the problem of right censoring because there would be very few participants with long follow-up times since first exposure.

## THE NECESSITY OF STUDIES WITH PREVALENT EXPOSURES

Studies of prevalent exposures are not only reasonable but also the best option in many situations. For many exposures, like smoking or other environmental or occupational exposures, it is unfeasible to start from the first exposure onward. For smoking, that would need follow-up from age 11 onward.

In situations where follow-up includes a mix of prevalent and incident exposures, as in many occupational studies, inclusion of prevalently exposed persons may greatly increase the statistical precision of the “late” exposure duration windows and/or the “long” time-since-first-exposure windows. This may produce lesser precision in the early time-since-first-exposure windows (5), but these are often of less interest.

Moreover, the situation is different for exposures that have temporary and current effects (e.g., oral contraceptives and venous thrombosis) versus exposures that have lasting effects (e.g., oral contraceptives and breast cancer). In the case of transient exposures, the problem might also be less important (cellular phone use and car accidents, coffee drinking and myocardial infarction).

Finally, it is often assumed that there are always potentially clearly demarked beginnings of an exposure. However, for many states, such as hypertension, hypercholesterolemia, dementia, diabetes, and so on, the exact date at which these conditions start is unknown. Nevertheless, these are clearly important etiological causes of disease.

## THE PROBLEM OF RIGHT CENSORING (MISSINGNESS ON THE RIGHT)

Although the problem of left truncation has received considerable attention, there has been relatively little discussion of the problem of right censoring. Ideally, every study should follow all participants until death, but this is rarely achievable. Unless there are unlimited resources and unlimited time available, any decision to restrict a study to incident users inevitably means restricting its size and restricting it to the earlier years since first exposure.

The problem of such restrictions is evident in the studies of cancer in atomic bomb survivors (49). An initial increase in leukemia and other hematological malignancies occurred, but the hazard ratio declined after about 10 years; in contrast, the increased risks for solid (epithelial) tumors only began to become apparent 10–15 years after exposure. Similar patterns were observed in studies of the development of secondary tumors after chemo- and radiotherapy for cancer, wherein randomized trials found little risk in contrast to observational long-term follow-up studies (50, 51).

Finally, other biases exist in observational studies, as well as randomized trials, that are due to self-selection toward exposures and self-selection in adherence to exposures. They can be important, but generally, they are similar between studies that start from first exposures and studies with prevalent exposures. Refer to Web Appendix 4.

## DISCUSSION

We have considered studies that enroll prevalently exposed persons (left truncation). The context of our discussion is the usefulness of dynamic populations with estimation of incidence rates and rate ratios for many environmental, occupational, and also pharmacoepidemiologic problems in cohort studies or case-control studies. It is apparent that the necessity of starting follow-up from first exposure should be decided on the basis of the best subject matter knowledge that is available.

If a more or less ideal cohort exists (large and extensive longitudinal data) with incident exposures and follow-up time in the *relevant* exposure time windows, this might be preferred. For all other situations, we will continue to need dynamic population studies, because either the ideal cohort does not exist or we cannot wait for it, or exposures are transient. A dynamic population study can be a cohort or a case-control study and is often the most feasible option; it needs information about only a few calendar years to yield data about many time-since-first-exposure windows. Even if a suitable cohort with incident exposures and very long-term follow-up exists, dynamic population studies can give more interesting insights, because the early as well as the late time-since-first-exposure experience has accrued over recent calendar times and has immediate relevance for the next calendar years.

Strong warnings about the inherent biasedness of studies involving prevalent exposures (5, 6) seem inconsistent with the practice and experience of epidemiology. Instead of demanding that studies start with newly exposed persons, it might be more thoughtful to discriminate among 3 situations:

1. Start of follow-up coincides with the beginning of exposure, which is alike to the randomized trial situation. This might often work in pharmacoepidemiology. A potential problem is that follow-up duration is usually short in such studies and does not allow the study of long-term risks.
2. There is a suitable mix of prevalent and incident exposures, that is, substantial numbers of new users as well as of prevalent users with different durations of exposure. This might often be the ideal situation, because it allows us to explore the effects of different durations of exposure (and/or time since first exposure) and greatly enhances information on long exposure durations. This is the case in many population-based case-control studies and occupational and environmental follow-up studies.
3. There are only persons with prevalent exposures. This makes it necessary to assess whether this might lead to completely wrong conclusions if there is a qualitative reversal of outcome between early and later time-since-first-exposure periods.

In situations 2 and 3, there is a need to consider whether the analyses should be stratified on time since first exposure, and whether the findings should be presented with this stratification. In addition, there should be caution in adjusting for confounders that may be consequences of previous exposure. All these considerations will be context specific as they will differ between different applications in epidemiology (e.g., between pharmacoepidemiology and occupational and environmental epidemiology), which often rely on data with different structures and different challenges. In many situations, such as on-off effects and transient exposures, this will not matter. No view from a single experience should become an absolute norm.

Finally, there are implications for teaching. All types of epidemiologic studies should continue to be taught. All designs have their indications and contraindications, and their merits differ in different situations. In particular, dynamic population studies, follow-up studies, and case-control studies will remain important epidemiologic tools and therefore should continue to be explicitly taught in introductory courses. Students of epidemiology should learn that the mere potential of a bias will not invalidate a study, but that in each case they should reason about whether the potential bias is likely to occur, how strong it is likely to be, and whether it can be removed by appropriate data analysis.

## Supplementary Material

Web Material
