# Supplementary material for: Point: Incident Exposures, Prevalent Exposures, and Causal Inference: Does Limiting Studies to Persons Who Are Followed From First Exposure Onward Damage Epidemiology?
Source: Am J Epidemiol. 2015 Oct 26;182(10):826–33. doi: 10.1093/aje/kwv225 (PMC4634310; doi:10.1093/aje/kwv225)
Supplement: Web Material [file supp_182_10_826__index.html]

Point: Incident Exposures, Prevalent Exposures, and Causal Inference: Does Limiting Studies to Persons Who Are Followed From First Exposure Onward Damage Epidemiology? — Web Material 

# Point: Incident Exposures, Prevalent Exposures, and Causal Inference: Does Limiting Studies to Persons Who Are Followed From First Exposure Onward Damage Epidemiology?

## Web Material

Web Material

- Web Material - Pdf file
