# Supplementary material for: Point: Incident Exposures, Prevalent Exposures, and Causal Inference: Does Limiting Studies to Persons Who Are Followed From First Exposure Onward Damage Epidemiology?
Source: Am J Epidemiol. 2015 Oct 26;182(10):826–33. doi: 10.1093/aje/kwv225 (PMC4634310; doi:10.1093/aje/kwv225)
Supplement: Web Material [file supp_kwv225_kwv225supp.pdf]

WEB MATERIAL

Accompanying:

Point: Incident Exposures, Prevalent Exposures, and Causal Inference: Does Limiting Studies to Persons Who Are Followed From First Exposure Onward Damage Epidemiology?

Jan Vandenbroucke[1] and Neil Pearce[2]

[1] Dr. Jan P Vandenbroucke [corresponding author]
Leiden University Medical Center
Dept. Clinical Epidemiology
PO Box 9600
2300 RC Leiden, The Netherlands
E-mail: J.P.Vandenbroucke@lumc.nl
Tel: +31-71-5264037; Fax: +31-71-5266994

[2] Dr. Neil Pearce,
Centre for Global NCDs
London School of Hygiene and Tropical Medicine
London, United Kingdom
and
Centre for Public Health Research
Massey University
Wellington, New Zealand

Web Appendix 1

The Protective Effect of Statins

It has been argued that observational studies of the expected beneficial effects of statin use suffer from bias due to enrolling prevalent users. In a systematic review wherein study results were classified by the enrollment of incident vs. prevalent users, studies with prevalent users showed an inordinately *large beneficial* effect, while studies with incident users showed results closer to those of randomized trials (1). The explanation may be a ‘healthy user effect’ because increasing ‘frailty’ leads to less preventive prescriptions and/or cessation of use (2). However, an in-depth attempt at emulating a randomized trial using observational data found the opposite: an *increased* mortality of current statin users which was mainly explained by time-varying confounding by indication for people with worse cardiovascular risk (3). Thus, the finding of increased mortality with current users in the latter study was the opposite of the findings from the systematic review in which current users had a decreased mortality. This demands an explanation as to why selection of current users might have such a different effect in these two publications (1, 3).

A reader of an earlier version of this manuscript suggested that the first group of studies mainly consisted of primary prevention studies, while the second reanalysis concerned a secondary prevention study. The different findings could then be due to different confounders in ‘primary’ vs. ‘secondary’ prevention studies. In our view, it is not certain how clear the distinction is between primary and secondary prevention in statin studies: the same cardiovascular risk factors, as well as other signs and symptoms of cardiovascular disease, will play a role in enrolment in both types of studies. When considering the literature, few ‘primary preventive’ studies are really ‘purely primary’, since in most of these studies (randomized trials as well as observational) persons are enrolled with quite strong existing cardiovascular risk factors and existing cardiovascular events in their medical history. The label ‘primary’ is used when, for example, the endpoint is myocardial infarction. In this situation, the persons enrolled have not yet

suffered a full blown myocardial infarction – yet persons are enrolled with a history of angina, a history of Transient Ischemic Attack or Cerebrovascular Accident, or other non-infarction coronary events. For example, the inclusion criteria of the PROSPER study (PROspective Study of Pravastatin in the Elderly at Risk)—often seen as one of the 'more primary prevention like'—were summarized as: "Patients were recruited if they had either preexisting vascular disease (coronary, cerebral, or peripheral) or were at increased risk for vascular disease due to such factors as smoking, hypertension, or diabetes. Inclusion criteria called for men and women between the ages of 70 and 82 years with a total plasma cholesterol of 155-350 mg/dL (4-9/mmol/L) and triglyceride levels < 200 mg/dL (6 mmol/L)." Source: http://www.medscape.com/viewarticle/444971.

It seems therefore uncertain whether the confounding in primary versus secondary prevention studies would be totally and strongly reversed in each other's opposite, so that 'current users' relative to non-users are totally different selections in these studies. Thus the proposed explanation seems incomplete. In general, pharmacoepidemiologic studies suggests that the main problem with assessing statin effects is increased frailty that leads to cessation of use or to non-use (2).

Web Appendix 2

Example of 'Stringing' Together Incidence Rate Ratios Over Follow-up Time

A case-control study of breast cancer irradiation and myocardial infarction (4) found an overall 7.4% increase in myocardial infarction per 1 Gray of radiation—irrespective of underlying risk cardiovascular risk factors. However, because this risk increment superimposes itself on the baseline risk, a life-table-like graphical presentation was necessary to convey the main message of the paper: that the absolute risk of irradiation increases with follow-up time and with baseline risk (See Web Figure 1). This presentation solved the problem of how to present the changes in effect of the same 7.4% increase during follow-up time, and corresponds to the plea to use more often life-table-like presentations when communicating the results of case-control studies (5).

Web Figure 1

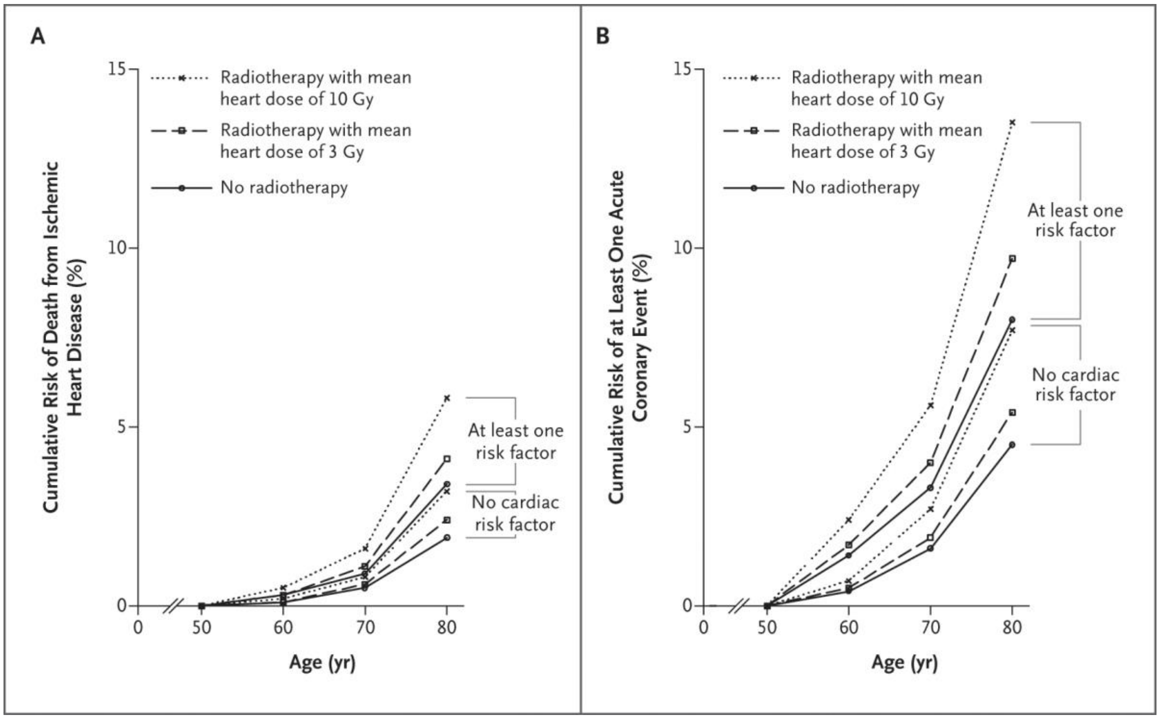

Web Appendix 3

Depletion of Susceptibles As an Explanation?

The situation of a complete reversal of hazard ratios on continued exposure as with cHRT and myocardial infarction, which was found in the Women's Health Initiative randomized trial, and later also shown in the observational reanalyses of the Nurses' Health Study and the observational part of the Women's Health Initiative (6, 7) is perhaps unusual and defies a simple explanation.

One proposed explanation is depletion of susceptibles (5). It seems unlikely that this is the complete explanation, since it does not seem to happen for any other disease whose incidence is influenced by cHRT. In particular, we would expect a similar effect with cHRT and venous thrombosis where also an initial strong peak of the hazard ratio was seen in randomized trials as well as observational studies, but this peak was then followed by a lower more or less constant, elevated hazard (8, 9). The early peak in venous thrombosis due to exogenous estrogens is generally explained by women who carry mutations that strongly increase the risk of venous thrombosis and whose effects are augmented by exogenous hormones (10). After that early peak, women still have an increased risk of developing venous thrombosis, and other known or unknown circumstances might be necessary to trigger the disease (for example, taking a long-haul flight, or after a plaster cast for a fracture, etc.).

It is difficult to imagine an early complete exhaustion of a pool of women who are *only* cardiovascularly susceptible to the effects of hormones, and *not* susceptible to any other exposures to develop myocardial infarction. An alternative possibility is that different mechanisms exist: some protective and others leading to increased risk.

In other instances, such as chronic exposures that lead to cancer, a complete reversal of an effect seems unlikely; even after cessation of exposure only a very gradual return to a background risk is expected.

Web Appendix 4

Other Selection and Self-Selection Problems in Observational Studies

All epidemiologic studies may suffer from self-selection of exposure (the type of person that starts a particular exposure) as well as self-selection in adherence to exposure (e.g. in pharmacoepidemiology). Difficult to define self-selection of exposure may make it difficult to define the comparator group. In principle, this problem is not different in first-exposure studies and in studies with prevalent exposures. In all types of studies one might need to assess differential adherence or differential loss to follow-up, and one might want to remedy these by statistical means such as imputation methods, marginal structural modeling, inverse probability weighting or related methods.

These selection problems are subject-matter specific. Pharmacoepidemiologists may encounter different problems than environmental or occupational epidemiologists. The 'first user cohort' principle was originally proposed in pharmacological contexts in which often immediate acute effects are studied, and where the sources of information are often different from occupational settings, and can be hugely different between health care systems. For instance, pharmacoepidemiology in the USA is challenged by a fragmented healthcare system wherein usually only some years of data are available on each person; so researchers cannot know with any certainty the duration of medication use, nor its beginning or its end. This might be very different in Scandinavian countries. The situaton might again be different from environmental or occupational exposures where employment with a certain factory has a fixed date, or moving to live to a house with increased radon exposure has a fixed date. This leads to the other advantage of occupational and environmental data: that one calendar time window can give information about different well-defined exposure windows (early, middle and longer duration).

References

1. Danaei G, Tavakkoli M, Hernán MA. Bias in observational studies of prevalent users: lessons for comparative effectiveness research from a meta-analysis of statins. *Am J Epidemiol.* 2012;175(4):250–262.
2. Glynn RJ, Schneeweiss S, Wang PS, Levin R, Avorn J. Selective prescribing led to overestimation of the benefits of lipid-lowering drugs. *J Clin Epidemiol.* 2006;59(8):819–828.
3. Danaei G, Rodríguez LA, Cantero OF, Logan R, Hernán MA. Observational data for comparative effectiveness research: an emulation of randomised trials of statins and primary prevention of coronary heart disease. *Stat Methods Med Res.* 2013;22(1):70–96.
4. Darby SC, Ewertz M, McGale P, Bennet AM, Blom-Goldman U, Bronnum D, et al. Risk of ischemic heart disease in women after radiotherapy for breast cancer. *N Engl J Med.* 2013;368(11):987–998.
5. Hernán MA. The hazards of hazard ratios. *Epidemiology.* 2010;21(1):13–15.
6. Prentice RL, Langer R, Stefanick ML, Howard BV, Pettinger M, Anderson G, et al. Combined postmenopausal hormone therapy and cardiovascular disease: toward resolving the discrepancy between observational studies and the Women's Health Initiative clinical trial. *Am J Epidemiol.* 2005;162(5):404–414.
7. Hernán MA, Alonso A, Logan R, Grodstein F, Michels KB, Willett WC, et al. Observational studies analyzed like randomized experiments: an application to postmenopausal hormone therapy and coronary heart disease. *Epidemiology.* 2008;19(6):766–779.
8. Cushman M, Kuller LH, Prentice R, Rodabough RJ, Psaty BM, Stafford RS, et al. Estrogen plus progestin and risk of venous thrombosis. *JAMA.* 2004;292(13):1573–1580.
9. Sweetland S, Beral V, Balkwill A, Liu B, Benson VS, Canonico M, et al. Venous thromboembolism risk in relation to use of different types of postmenopausal hormone therapy in a large prospective study. *J Thromb Haemost.* 2012;10(11):2277–2286.
10. Bloemenkamp KW, Rosendaal FR, Helmerhorst FM, Vandenbroucke JP. Higher risk of venous thrombosis during early use of oral contraceptives in women with inherited clotting defects. *Arch Intern Med.* 2000;160(1):49–52.
